# Chemoenzymatic one-pot reaction from carboxylic acid to nitrile *via* oxime†

**DOI:** 10.1039/d1cy01694f

**Published:** 2021-11-30

**Authors:** Melissa Horvat, Victoria Weilch, Robert Rädisch, Sebastian Hecko, Astrid Schiefer, Florian Rudroff, Birgit Wilding, Norbert Klempier, Miroslav Pátek, Ludmila Martínková, Margit Winkler

**Affiliations:** Institute of Molecular Biotechnology, Graz University of Technology Petersgasse 14 A-8010 Graz Austria margit.winkler@tugraz.at; Institute of Microbiology of the Czech Academy of Sciences Vídeňská 1083 CZ-142 20 Prague Czech Republic martinko@biomed.cas.cz; Department of Genetics and Microbiology, Faculty of Science, Charles University Viničná 5 CZ-12844 Prague 2 Czech Republic; Institute of Applied Synthetic Chemistry, TU Wien Getreidemarkt 9/OC-163 A-1060 Vienna Austria; Acib GmbH Krenngasse 37 A-8010 Graz Austria; Institute of Organic Chemistry, Graz University of Technology Stremayrgasse 9 A-8010 Graz Austria

## Abstract

We report a new chemoenzymatic cascade starting with aldehyde synthesis by carboxylic acid reductase (CAR) followed by chemical *in situ* oxime formation. The final step to the nitrile is catalyzed by aldoxime dehydratase (Oxd). Full conversions of phenylacetic acid and hexanoic acid were achieved in a two-phase mode.

## Abstract 2

We report a new chemoenzymatic cascade starting with aldehyde synthesis by carboxylic acid reductase (CAR) followed by chemical *in situ* oxime formation and enzymatic dehydration by aldoxime dehydratase (Oxd).

## Graphical abstract

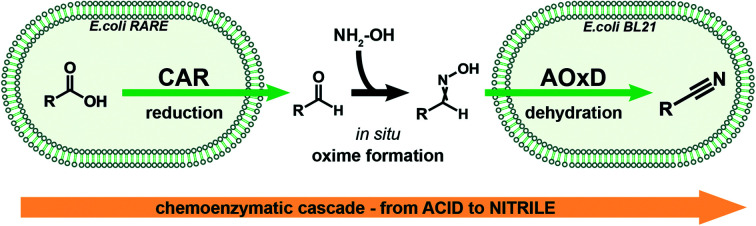

The nitrile functionality is versatile.^1^ Nitriles serve as building blocks for a range of chemical transformations *e.g.* hydrolysis to acids, hydration to amides, reduction to amines, cyclization to N-heterocyclic compounds and many others. In conventional organic synthesis, nitriles may be synthesized by addition of cyanide or substitution of leaving groups by cyanide. Other applied reactions use transition metal-catalyzed cyanation and electrophilic cyanide transfer and involve highly toxic cyanide.^2^ The carbon chain is extended by one C atom in these reactions (Scheme 1A).^3^ Avoiding the use of cyanide in nitrile synthesis is an important step in replacing hazardous and highly toxic chemicals. Cyanide-free procedures for nitrile syntheses from aldehydes, amines, azides, oximes, halides and arenes have therefore been established.^2,4^

**Scheme 1:**
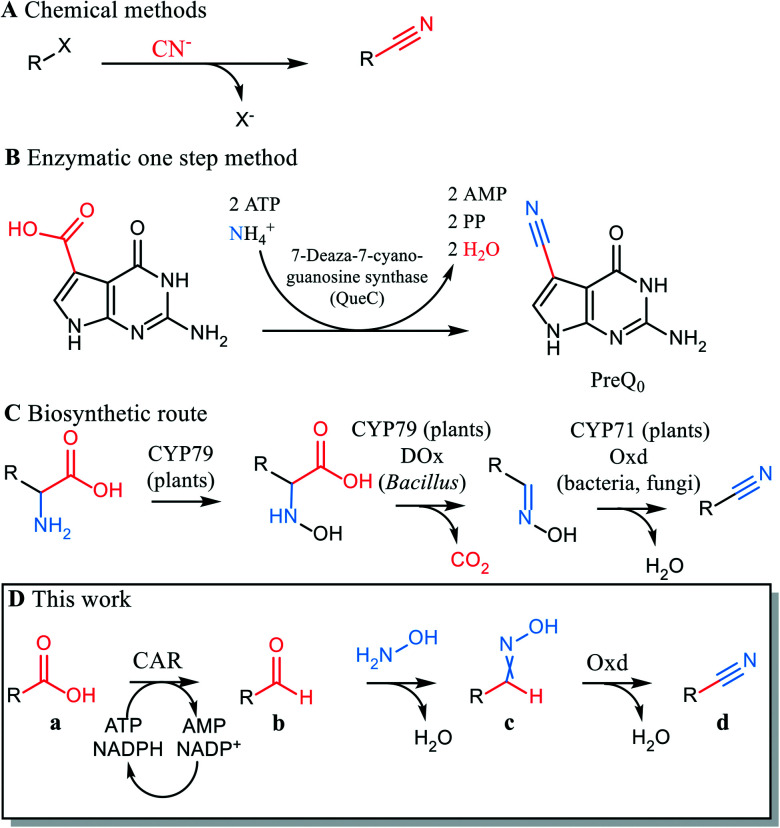
Routes from acid to nitrile. A: Substitution of leaving group by cyanide. Carbon chain is extended; B: acid carbon becomes nitrile carbon. Carbon chain length is retained; C: acid carbon is released as CO_2_. Carbon chain length is reduced; CYP79 = cytochrome P450 (CYP79 family), CYP71 = cytochrome P450 (CYP71 family), DOx = decarboxylase/oxidase, Oxd = aldoxime dehydratase; D: chemoenzymatic route. CAR = carboxylic acid reductase. Carbon chain length is retained.

Carboxylic acids are abundant in biomass and ways to replace current methods that start from fossil precursors are sought after. A classical route from carboxylic acids to nitriles consists of acid activation followed by amide formation and chemical dehydration with phosphoric acid at high temperature. These conditions are only compatible with simple, unfunctionalized molecules, and not appropriate *e.g.* for late stage modifications. Recently, a single step reaction from acid to nitrile was reported that involves light-driven decarboxylation and cyanation of an acid in the presence of stoichiometric amounts of cyanobenziodoxolones. CO_2_ is eliminated and cyanide introduced in this synthetic sequence.^5^

The direct conversion of a carboxylic acid to the respective nitrile was described in the biosynthesis pathway of deazapurine-containing compounds such as the hypermodified tRNA bases queuosine^6^ and archaeosine^7^ as well as the nucleoside antibiotics toyocamycin^8^ and sangivamycin.^9^ The reaction is catalyzed by 7-cyano-7-deazaguanine synthase (EC 6.3.4.20, QueC), which activates the carboxylate at the expense of ATP. Ammonia acts as a nucleophile,^9^ and an intermediate amide reacts with a second ATP to give the nitrile.^10^ The conceptual elegance of this enzymatic reaction cannot be exploited for synthesis, because QueC synthases are strictly specific for their natural substrate^11^ (Scheme 1B). Of biogenic nitriles other than the deazanucleoside preQ_0_, phenylacetonitrile is derived from amino acids in the bacterium *Bacillus* sp. OxB-1 through a decarboxylase/oxidase (DOx) – aldoxime dehydratase (Oxd) pathway (Scheme 1C).^12^ In this case, CO_2_ is released, and the carbon chain of the substrate shortened. The Oxd (EC 4.99.1.5-7) in this reaction sequence is a heme-protein that catalyzes oxime dehydration. Oxds were found in the bacteria *Rhodococcus*, *Pseudomonas*, *Bradyrhizobium* and in fungi (*Fusarium*, *Sclerotinia*)^13–15^ and have been studied as catalysts for mild and sustainable nitrile synthesis.^16,17^ Their biocatalytic potential lies in their ability to dehydrate oximes in an aqueous medium and thus produce nitriles in a cyanide-free fashion. Oxds were shown to react with aliphatic and (chiral) aryl aliphatic oximes,^18,19^ oximes attached to alicycles,^20^ dioximes^17^ and benzisoxazoles^21^ (*de facto* circular oximes) in a Kemp-type elimination.^22^ However, little or no activity of Oxds were found for substrates with the oxime moiety directly attached to a (hetero)aromatic ring.^13^

We report here a new artificial pathway from acid to nitrile (Scheme 1D), in which the carbon atom of the carboxylic acid eventually becomes the nitrile carbon and thus, the carbon chain length is retained. The proposed cascade includes two enzymatic steps and one chemical reaction. In the first step, a carboxylic acid reductase (EC 1.2.1.30, CAR) reduces the carboxylate to the respective aldehyde. In the intermediate chemical step, the aldehyde is trapped by oximation with hydroxylamine. The resultant aldoxime finally undergoes enzymatic dehydration mediated by Oxd (Scheme 1D).

The first step in the envisaged cascade reaction was a CAR-mediated acid reduction to the respective aldehyde in a whole cell system. In this setup, oxygen is required for cell viability and constant ATP formation, the cofactor needed for carboxylate activation. The presence of hydroxylamine in the reaction medium should lead to chemical scavenging of the aldehyde as an aldoxime, the substrate for the second enzymatic (dehydration) step. The majority of Oxds were highly active only under anaerobic conditions, which maintained the catalytically essential ferrous state of the heme. The enzyme OxdBr1 from *Bradyrhizobium* sp. LTSPM299 is an exception, since it does not require strictly anaerobic conditions.^15^ This fact was important in light of the oxygen requirement of the CAR-containing living cell biocatalyst and rendered OxdBr1 a promising candidate for the cascade.

In contrast to CARs with their exceptionally broad substrate tolerance,^25^ little was known about OxdBr1 activities for substrates other than (aryl)aliphatic aldoximes. Therefore, we began the development of the reaction setup by searching for compounds compatible with both biocatalysts. Whole-cell reactions mediated by OxdBr1 with structurally diverse *E*/*Z*-aldoximes achieved the highest conversions for phenylacetaldehyde oxime 1c and hexanal oxime 5c, followed by medium conversions of cinnamaldehyde oxime 4c and vanillin oxime 2c as analogues of typical CAR products (Table 1).^25^ A low conversion was found for piperonal oxime 3c. The same trend was observed when OxdBr1 was applied as partly purified enzyme (ESI,† Fig. S1A and B). Whereas oximes 1c and 5c are usually well accepted by other Oxds,^13^ oximes 2c, 3c, 4c have not yet been explored, which broadens the range of potential Oxds uses. In this work, the best substrates 1a and 5a were selected to examine the proposed pathway. The CAR from *Neurospora crassa* is one of the most active CARs reported for 5a reduction (Scheme 2),^26,27^ and was selected for the whole cell-mediated aldehyde synthesis step of the cascade.

**Table 1:** Substrate scope of aldoxime dehydratase OxdBr1

	Substrate	Conv.^a^ [%]	Anal. yield of nitrile **d** [mM]
1c^b^	Phenylacetaldehyde oxime	93.2 ± 1.5	9.40 ± 0.16
2c	Vanillin oxime	25.7 ± 1.5	1.28 ± 0.08
3c^b^	Piperonal oxime	1.9 ± 0.3	0.11 ± 0.02
4c^b^	Cinnamaldehyde oxime	32.3 ± 4.9	2.66 ± 0.41
5c^b^	Hexanal oxime	100.0 ± 2.7	5.89 ± 0.17

a Single-phase whole cell reaction, OD10, HEPES pH 7.5, 1 h, 28 °C, 10 mM of **c**.

b Synthesized according to literature as described in ESI.†^23,24^

**Scheme 2:**
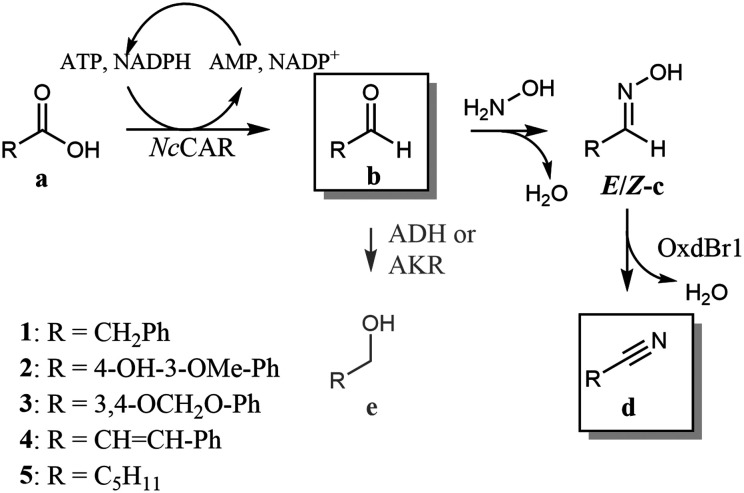
Chemoenzymatic cascade from carboxylic acid to nitrile. Compounds in frames are predominantly present in the organic phase when the reaction is performed in two-phase mode.

The metabolic activity of CAR-expressing *E. coli* cells needs to be high to ensure efficient ATP and NADPH supply. Therefore, the cytotoxic aldehyde should be eliminated using, *e.g.*, a follow-up reaction^28–30^ or *in situ* product removal (ISPR).^26,31^ In this work, the aldehyde is removed by follow-up reaction as an oxime. However, in solely aqueous systems, aliphatic and highly volatile compounds were particularly challenging to handle in terms of mass balance (see Table 1, entry 5c). Therefore, we took advantage of ISPR as additional means to improve product recovery. We previously reported that CAR biocatalysts retain their activity, when exposed to water-immiscible organic solvents like *n*-heptane or biocompatible *n*-hexadecane for ISPR,^26,27^ but little was known about co-solvent effects of these particular solvents on Oxds. Previous studies with different Oxds reported on (di)nitrile products as second layer to achieve exceptional product titers in oxime dehydration reactions,^32^ which hints towards organic solvent tolerance of Oxds.^32^ To verify this assumption, immiscible organic solvents previously selected for aldehyde production^26^ were examined as media in the oxime dehydration step. At the same time, the robustness of OxdBr1 towards oxygen was assessed because the reactions were carried out in tubes with a large headspace of air. Full conversion of 5c was observed under these conditions, indicating no significant inhibition by oxygen. Small amounts of nitrile in control reactions were the result of thermal dehydration of the oxime in the GC injector. These did not occur when reactions were analyzed by HPLC. Mass balance was greatly enhanced when *n*-heptane or *n*-hexadecane were applied for the formation of highly volatile 5d (Fig. 1). The use of nitrile products as second phase would constitute the most elegant solvent. While this is feasible for Oxd reactions, it is expected to be problematic for the carboxylic acid reduction step because CARs are inhibited by 1d (ESI,† Fig. S3).

**Fig. 1:**
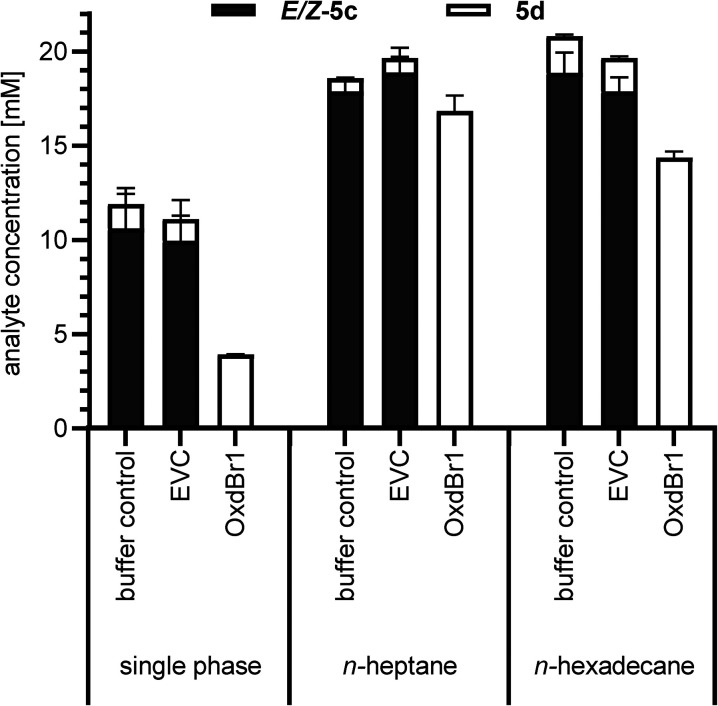
Comparison of OxdBr1 catalyzed dehydration of hexanal oxime (5c, 20 mM) in single-phase (aqueous) and two-phase (*n*-heptane/aqueous or *n*-hexadecane/aqueous) mode. Whole cells, OD10; 4 h, 28 °C. EVC = empty vector control. Error bars are shown for technical triplicates.

Scheme 2 summarizes the chemoenzymatic cascade reaction from acid to nitrile. Having established an overlapping substrate profile and explored the presence of oxygen and organic solvents, we considered further critical reaction parameters, like pH value and hydroxylamine concentration. The rate of oxime formation is particularly high at very acidic or basic conditions and hydroxylamine in excess (50-fold and more).^33^ However, these conditions might be critical 1) in view of cell viability and 2) in view of inhibition phenomena.

The pH of *in situ* oxime formation is predetermined by the living cell catalyst's preference. Aldehyde formation by CAR enzymes has been described in a broad pH range. We opted for pH 6.5, because living *E. coli* cells can cope with a pH of 6.5^27^ and hydroxylamine was reported to react with aldehydes and ketones under these conditions to form aldoximes and ketoximes, respectively.^34^

High hydroxylamine concentrations to push aldehyde conversion to oximes were expected to be problematic.^35–39^ We used a moderate molar excess (1.5-fold) of hydroxylamine compared to carboxylic acid substrate.

The cascade reactions (Scheme 2) were studied in two different reaction setups. In the first approach, a one-pot cascade was performed with a mix of *E. coli* whole cells expressing *Nc*CAR and others expressing OxdBr1 in the presence of **a** (10 mM), and hydroxylamine (1.5 eq.) (one-pot, Fig. 2). All constituents were incubated in a two-phase system (aqueous/*n*-heptane 1/1) for 24 h at 28 °C. Fig. 2 shows nitrile formation. Phenylacetic acid 1a was fully reduced to aldehyde 1b. The intermediate oxime formation was incomplete, as judged by remaining aldehyde. The enzymatic dehydration step, on the other hand, was highly efficient, as oxime was only detected in trace amounts. Due to incomplete oxime formation, aldehyde was partly over-reduced to alcohol 1e. The resulting composition in case of the aliphatic acid 5a was less on the product side. In this case, neither acid reduction, oxime formation nor dehydration went to completion. Not surprisingly, also over-reduction was more prominent in case of this short chain fatty acid.^26^ The one-pot reactions resulted in 4.6 mM of nitrile 1d or 3.8 mM of nitrile 5d, respectively.

**Fig. 2:**
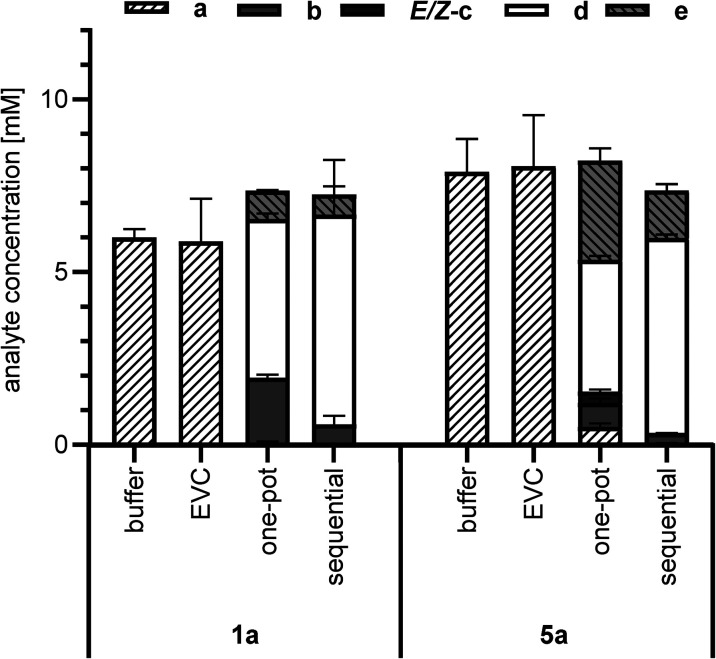
Chemoenzymatic cascade from carboxylic acid (**a**) to nitrile (**d**) in biphasic system (aqueous/*n*-heptane 1/1). Whole cell-mediated reduction of 1a or 5a using *Nc*CAR, *in situ* oximation by hydroxylamine (1.5 eq.) and enzymatic dehydration to 1d or 5d by OxdBr1 in whole cells. One-pot: mixed culture of cells harbouring either *Nc*CAR or OxdBr1. Time: 24 h. Sequential: first, *Nc*CAR harbouring cells were incubated with hydroxylamine for 4 h. Second, OxdBr1 harbouring cells were added and reactions were stopped after 24 h. 10 mM substrate was dissolved in *n*-heptane; temp.: 28 °C. Error bars are shown for technical triplicates. EVC = empty vector control.

In the second approach (sequential, Fig. 2), the cascade was divided into two subsequent steps. *In vivo* bioreduction and *in situ* oxime formation were performed during the first 4 h. Without any work-up, the resulting mixture was supplemented with OxdBr1 biocatalyst. Full consumption of both 1a and 5a was detected, but approximately 20% of the added material was not recovered. Of the recovered product mixture, 83% was the desired aryl-aliphatic product 1d in case of 1a. In case of 5a, 76% was nitrile 5d and the major side product was 5e. In total, nitrile titers were higher in these sequential two-step/one-pot reactions (6.1 mM 1d and 5.7 mM 5d, respectively) as compared to the simultaneous one-pot reactions (Fig. 2). One of the reasons might be that the *Nc*CAR was inhibited by **d**, which only emerges in the final reaction step.

With 1a in a concentration of 20 mM, full substrate consumption was again achieved in the one-pot two-step mode. After 24 h, 15.2 mM (76% yield) of nitrile 1d were detected, in addition to 0.4 mM of 1b, 2.3 mM of ***E*** and ***Z*-**1c and 1.4 mM of 1e (Scheme 3).

**Scheme 3:**
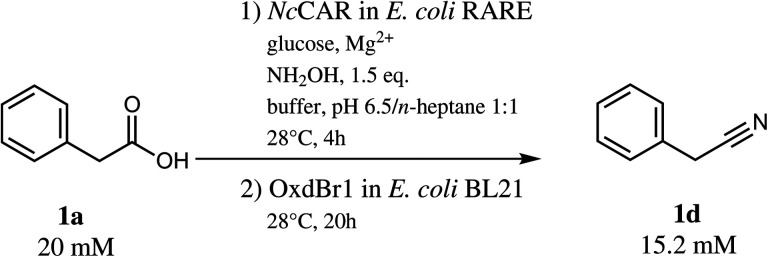
One-pot two-step transformation of phenylacetic acid (1a) to phenylacetonitrile (1d).

These results are a promising proof of the cascade concept and the starting point for further in-depth investigation, including diversification of the product range by tailored combinations of various CARs and Oxds.

## Conclusions

In summary, we showed a chemoenzymatic route from carboxylic acid to nitrile in which the carbon-chain length of the starting material is retained. For this approach, a three-step cascade was used. In the first step, *E. coli* whole cell catalysts co-expressing CAR and PPTase reduced the carboxylic acid to the respective aldehyde. Hydroxylamine trapped this cytotoxic compound to give aldoxime in a chemical step. Thirdly, the intermediate aldoxime was enzymatically dehydrated to the desired nitrile by Oxd. Up to 76% of target product was obtained in a biphasic system. The organic layer was beneficial for the recovery rate, because it retained the lipophilic volatiles which would otherwise escape to the gas phase. The organic phase did not affect OxdBr1 performance. The proposed route is environmentally more benign since it does not require cyanide salts, toxic metals, or undesired oxidants in contrast to entirely chemical procedures. Future in depth-studies will extend this proof of concept by examination of various CAR and Oxd enzymes to target a wider variety of nitriles.

## Author contributions

MP, LM and MW: conceptualization; MH, VW, RR, SH, AS, BW: investigation and methodology; MH, MW, SH, BW: formal analysis; MH, MW: visualization, data curation; FR, NK, MP, LM, MW: supervision; MP, LM, MW: funding acquisition; MH, MW: writing – original draft. FR, BW, MP, LM: writing – review & editing.

## Conflicts of interest

There are no conflicts to declare.

## Supplementary Material

CY-012-D1CY01694F-s001
