# Supplementary material for: Chemoenzymatic one-pot reaction from carboxylic acid to nitrile via oxime
Source: Catal Sci Technol. 2021 Nov 30;12(1):62–6. doi: 10.1039/d1cy01694f (PMC8725990; doi:10.1039/d1cy01694f)
Supplement: CY-012-D1CY01694F-s001 [file CY-012-D1CY01694F-s001.pdf]

Electronic Supplementary Material (ESI) for Catalysis Science & Technology.
This journal is © The Royal Society of Chemistry 2021

Supplementary information

Chemoenzymatic one-pot reaction from carboxylic acid to nitrile via oxime

Melissa Horvat[a], Victoria Weilch[a], Robert Rädisch[b,c], Sebastian Hecko[d], Astrid Schiefer[d], Florian Rudroff[d], Birgit Wilding[e,f], Norbert Klempier[f], Miroslav Pátek[b], Ludmila Martínková[b]*, Margit Winkler [a,e]*

[a] Institute of Molecular Biotechnology, Graz University of Technology, Petersgasse 14, A-8010 Graz, Austria. E-mail: margit.winkler@tugraz.at
[b] Institute of Microbiology of the Czech Academy of Sciences, Vídeňská 1083, CZ-142 20 Prague, Czech Republic. E-mail: martinko@biomed.cas.cz
[c] Department of Genetics and Microbiology, Faculty of Science, Charles University, Viničná 5, CZ-12844 Prague 2, Czech Republic.
[d] Institute of Applied Synthetic Chemistry, TU Wien, Getreidemarkt 9/OC-163, A-1060 Vienna, Austria
[e] acib GmbH, Krenngasse 37, A-8010 Graz, Austria
[f] Institute of Organic Chemistry, Graz University of Technology, Stremayrgasse 9, A-8010 Graz, Austria

Contents

1. Additional data and figures 3

1.1 Substrate scope of enriched OxdBr1 3

1.2 *In situ* oxime formation in two-phase system 4

1.3 Effects of ***E/Z*-1c** or **1d** on specific activity of purified *Nc*CAR 4

2. Materials and Methods 5

2.1 General 5

2.2 Strains and cultivations 5

2.3 Enriched OxdBr1 5

2.4 Substrate scope with enriched OxdBr1 in single-phase mode 5

2.5 Whole cell-mediated biotransformations with OxdBr1 in two-phase mode 5

2.6 Specific activity analysis of *Nc*CAR with ***E/Z*-1c** and **1d** 5

2.7 *In situ* oxime formation in biphasic system 6

2.8 Chemoenzymatic cascade with *Nc*CAR and OxdBr1 6

2.9 GC-FID analysis for compounds **1** and **5** 6

2.10 HPLC-UV analysis of compounds **2**, **3** and **4** 6

2.11 Phenylacetaldehyde oxime (**1c**) synthesis and purification 6

2.12 Piperonal oxime (**2c**) synthesis and purification 7

2.13 Cinnamaldehyde oxime (**4c**) synthesis and purification 8

2.14 Hexanal oxime (**5c**) synthesis and purification 8

3. References 9

1. Additional data and figures

1.1 Substrate scope of enriched OxdBr1

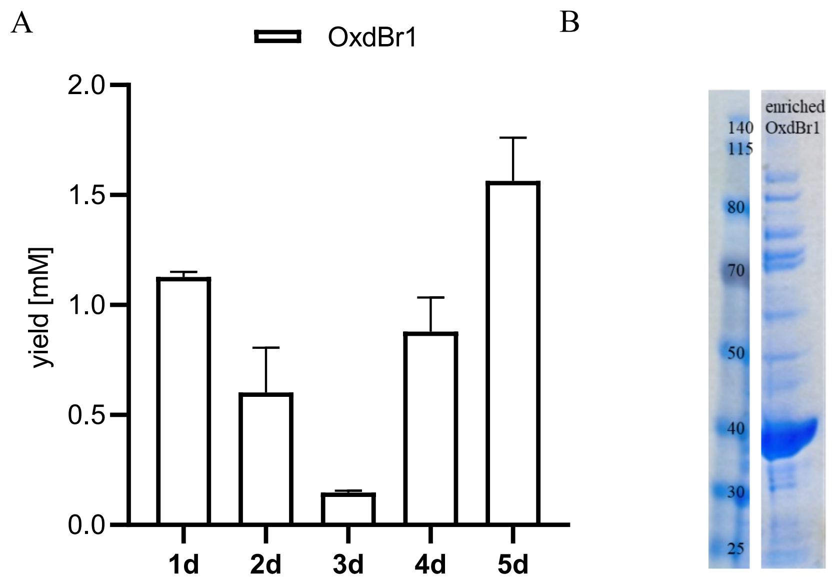

Figure S1A. Nitrile formation with OxdBr1 (partly purified). *In vitro* dehydration using phenylacetaldehyde oxime (**1c**), vanillin oxime (**2c**), piperonal oxime (**3c**), cinnamaldehyde oxime (**4c**) and hexanal oxime (**5c**) as substrates (10 mM each); 10µg enriched enzyme, vol: 500 µL, time: 1 h, temp.:28°C. Error bars are shown for technical triplicates. **B.** SDS-PAGE with enriched OxdBr1 samples. 5 µg of enzyme preparation were loaded. Prestained protein ladder was used as standard.

1.2 In situ oxime formation in two-phase system

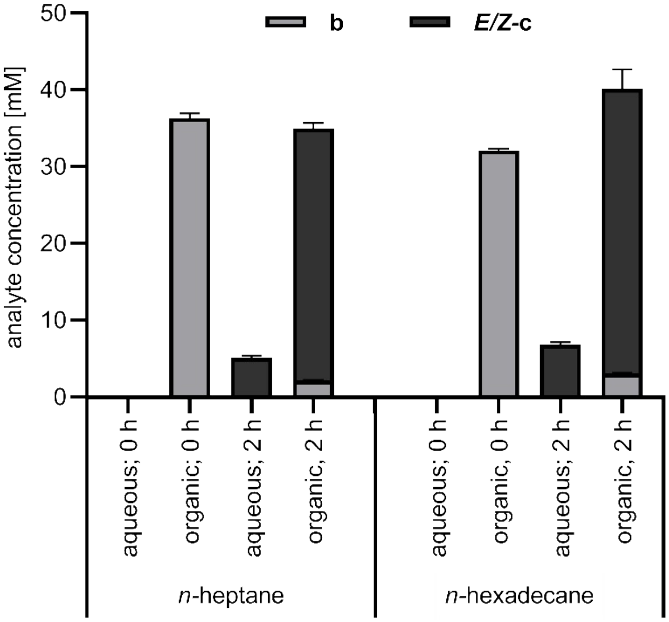

Figure S2. *In situ* oximation using hexanal (**5b**) as substrate to form (**5c)** in biphasic system. 40 mM substrate were dissolved in either *n*-heptane or *n*-hexadecane. 48 mM hydroxylamine was added to the aqueous phase. Time: 2 h, temp.:28°C. Error bars are shown for technical triplicates.

1.3 Effects of **E/Z-1c** *or* **1d** *on specific activity of purified NcCAR*

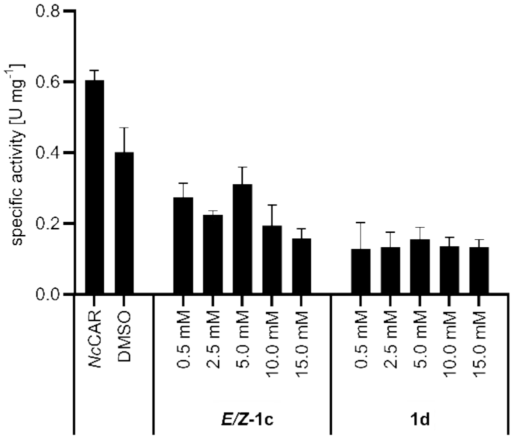

Figure S3. Effects of 0.5-15 mM of ***E/Z*-1c** or **1d** on specific activities of partially purified *Nc*CAR. **1a** was used as substrate for NADPH-depletion monitoring at 340 nm. **1c** or **1d** were added dissolved in DMSO. As a control, pure DMSO was added in the same amount. Error bars show the standard deviation of three technical replicates. One unit is defined as the amount of enzyme preparation catalyzing the oxidation of 1 µM NADPH per minute. In blank reactions (not shown), ATP was omitted.

2. Materials and Methods

2.1 General

Compounds, if not otherwise stated, were purchased from Sigma Aldrich (Vienna, Austria) with the highest available purities.

2.2 Strains and cultivations

OxdBr1 expressed in *E. coli* BL21 Star™ (DE3) was cultivated as described previously.[1] *E. coli* K-12 MG1655 RARE (DE3) expressing *Nc*CAR was cultivated according to Schwendenwein *et al.*[2] The empty vector control (EVC) used for the dehydration reactions was pET28a(+) whereas the pETDuet-1:*Ec*PPTase was used for the cascade reactions.

2.3 Enriched OxdBr1

Cell disruption and Ni-affinity chromatography of OxdBr1 for enriched enzyme preparation were conducted as described in Horvat *et al.*[3]

2.4 Substrate scope with enriched OxdBr1 in single-phase mode

In vitro dehydrations were performed with 10 µg of enriched OxdBr1 in 300 mM HEPES buffer, pH 7.5. Substrate (25 µL, 200 mM in DMSO) was added to give a 10 mM substrate solution. 500 µL dehydration reactions were performed at 28°C for 1 h and 900 rpm in an Eppendorf thermomixer. *In vitro* conversions with compound **2c** were terminated by addition of 1000 µl of EtOH. Conversions with compounds **3c** and **4c** were terminated by addition of 1000 µl of MeOH. Samples were vortexted, centrifuged for 20 min at 13,200 rpm and then analyzed by HPLC. Biotransformations with substrates **1c** and **5c** were extracted with 2x 500 µL of ethyl acetate and 0.01% internal standard (IS), dried over Na_2SO_4 and analyzed by GC- FID. Technical triplicates were determined and are represented by error bars.

2.5 Whole cell-mediated biotransformations with OxdBr1 in two-phase mode

Biodehydrations were performed with 10 OD_{600} units [approx. 10 mg of cell wet weight (CWW) mL^{-1}] of *E. coli* BL21 Star™ (DE3) expressing OxdBr1 in 1 mL of 300 mM HEPES buffer, pH 7.5. Substrate was dissolved in 1 mL of *n*-heptane with 0.01% internal standard (IS, *n*-tetradecane), *n*-hexadecane with 0.01% IS (*n*-heptane) or dissolved in DMSO and added to the aqueous phase (5% DMSO). Biotransformations were performed at 28°C for either 1 h or 4 h. Reactions with **2c** were terminated by the addition of EtOH and those with **3c** and **4c** were stopped by the addition of 1000µl of MeOH**.** Samples were vortexted and centrifuged for 20 min at 13,200 rpm. Supernatants were analyzed by HPLC-UV. Reactions with **1c** and **5c** were extracted with 2x 1 mL of ethyl acetate and 0.01% internal standard or the organic layer was directly dried over Na_2SO_4 and analyzed with GC-FID.

2.6 Specific activity analysis of NcCAR with **E/Z-1c** *and* ***1d***

NADPH-depletion monitoring at 340 nm was used to determine specific activities of IMAC-purified *Nc*CAR. Purification and assay procedures were performed as described.[2] **1a** was used as substrate, ***E*/*Z*-1c** and **1d** were dissolved in DMSO and added to the reaction mix to final concentrations of 0.5-15mM.

2.7 In situ oxime formation in biphasic system

For oxime formation reactions, 48 mM NH_2OH in the aqueous phase (500 µl of 300 mM HEPES, pH 7.5) was overlayed with the same volume of water-immiscible solvent containing 40 mM **5b** and 0.01% of *n*-tetradecane (IS). The reaction mixture was incubated at 28°C for 2 h and 320 rpm. The aqueous phase before and after incubation was extracted twice with 500 µL of ethyl acetate with 0.01% of *n*-tetradecane. Organic layers were dried over Na_2SO_4 and analyzed by GC-FID.

2.8 Chemoenzymatic cascade with NcCAR and OxdBr1

Two-phase cascade reactions were carried out as follows: The organic layer contained carboxylic acid **1a** or **5a** (10 mM) dissolved in *n*-heptane (1 mL, 0.01% IS). The aqueous phase consisted of 1 mL MES buffer (300 mM, pH 6.5) with glucose (100 mM), $MgSO_4$ (71 mM) and NH_2OH (15 mM). For the first step of the cascade (enzymatic reduction), 20 OD_{600} units (approx. 20 mg of CWW mL^{-1}) of *E. coli* MG1655 RARE (DE3) cells expressing *Nc*CAR were used. For the biodehydration of **1c** or **5c** to **1d** or **5d**, resp., 10 OD_{600} units (approx. 10 mg of CWW mL^{-1}) of *E. coli* Star™ BL21 (DE3) expressing OxdBr1 were added.

One pot reaction: Cells expressing either *Nc*CAR or OxdBr1 were simultaneously incubated with hydroxylamine for 24 h.

Sequential reaction: *E. coli* cells expressing *Nc*CAR were incubated with hydroxylamine for 4 h before *E. coli* cells expressing OxdBr1 were added. The cell mixture was incubated for additional 20 h. The reactions proceeded at 28°C and were terminated with 3 M HCl after 24 h. The *n*-heptane phase was dried over Na_2SO_4 and analyzed by GC-FID.

*2.9 GC-FID analysis for compounds **1** and **5***

For GC-FID measurements, a ZP-5 column (crosslinked 5% Ph-Me Siloxane; 30 m, 0.32 mm diameter, 0.25 µm film thickness) on a Shimadzu GC 2030 equipped with an FID was used. Sample aliquots of 1 µL were injected in split mode (split ratio 10:1) at 240 °C injector temperature and 320 °C detector temperature with N_2 as carrier gas. The temperature program for quantification of **1a** and derivatives was as follows: hold at 70 °C for 3 min, followed by a temperature gradient to 150 °C at 20 °C min^{-1} and a hold at 150 °C for 5 min, continued by a temperature ramp to 300 °C at 40 °C min^{-1} for 3 min. The total runtime for **1a/1b/*E*/*Z*-1c/1d/1e** was 18.75 min. The temperature gradient for **5a/5b/ *E*/*Z*-5c/5d/5e** started with a hold at 70 °C for 14 min, followed by a temperature gradient to 300 °C at 40 °C min^{-1} and a hold at 300 °C for 2 min. The total run time was 21.75 min. GC-FID results were evaluated with the GC-FID Data Analysis software LabSolution (Shimadzu). Quantification of all compounds was established through linear interpolation from calibration curves with authentic standard.

*2.10 HPLC-UV analysis of compounds **2**, **3** and **4***

HPLC-UV analysis for compounds **2**, **3** and derivatives were performed according to the methods previously reported.[4,5] The analytical method for compound **4** and derivatives was described by Schwendenwein *et al.*[2]

*2.11 Phenylacetaldehyde oxime (**1c**) synthesis and purification*

A round bottom flask (RBF) (250 mL) was charged with sodium carbonate (8.15 g, 76.93 mmol, 2.00 equiv.). Sodium carbonate was dissolved in water (25 mL) and ethanol (35 mL) and hydroxylamine hydrochloride (5.40 g, 76.93 mmol, 2.00 equiv.) and **1b** (5.00 mL, 38.46 mmol, 1.00 equiv.) were added. The reaction was stirred for 19 h at 50°C. Thin layer chromatography (TLC) analysis [cyclohexane (CY):

ethyl acetate (EA), 9:1, UV detection] confirmed full conversion. The reaction was diluted with deionized water and extracted with dichloromethane (5 x 100 mL). The combined organic layers were dried over Na_2SO_4 and concentrated. The crude product was purified by flash chromatography (CH_2Cl_2 + 1 % MeOH, 185 g silica gel) to yield 1.05 g of product with an *E*/*Z*-ratio of ~1/1.4.

*2.12 Piperonal oxime (**2c**) synthesis and purification*

A RBF (250 mL) was charged with sodium carbonate (3.72 g, 35.0 mmol, 0.75 equiv.). The sodium carbonate was dissolved in water (95 mL) and ethanol (5 mL) and hydroxylamine hydrochloride (5.03 g, 72.0 mmol, 1.50 equiv.) and **2b** (7.01 g, 46.7 mmol, 1.00 equiv.) were added. The reaction mixture was stirred for 17 h at rt during which precipitation of the product occurred. TLC analysis [light petroleum (LP): EA = 5:1, stained with $KMnO_4$] of the reaction mixture confirmed full conversion. The suspension was poured onto ice-water (about 100 mL) and the solids were separated via filtration. The obtained colorless solids were washed thoroughly with water (3 x 20 mL) and then recrystallized from toluene to yield 5.48 g (71 %) of the desired piperonal oxime as colorless crystals with an *E*/*Z*-ratio of 94/6.

^{1}H NMR (600 MHz, Chloroform-*d*) δ 5.99 (s, 2H, *E*-H2), 6.02 (s, 2H, *Z*-H2), 6.81 (d, *J* = 8.0 Hz, 1H, *E*-H4), 6.86 (d, *J* = 8.1 Hz, 1H, *Z*-H4), 6.96 (dd, *J* = 8.0, 1.7 Hz, 1H, *E*-H5), 7.16 (d, *J* = 1.6 Hz, 1H, *E*-H7), 7.27 – 7.31 (m, 1H, *Z*-H5), 7.73 (d, *J* = 1.7 Hz, 1H, *Z*-H7), 8.05 (s, 1H, *E*-H8).

^{13}C NMR (151 MHz, Chloroform-*d*) δ 101.6 (*E*-C2), 105.8 (*E*-H7), 108.5 (*E*-C4),123.1 (*E*-H5), 126.4 (*E*-C6), 148.4 (*E*-C3a/*E*-C7a), 149.4 (*E*-C3a/*E*-C7a), 150.1 (*E*-C8). (only the *E*-isomer was assigned)

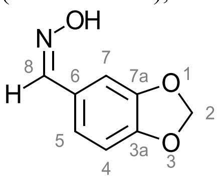

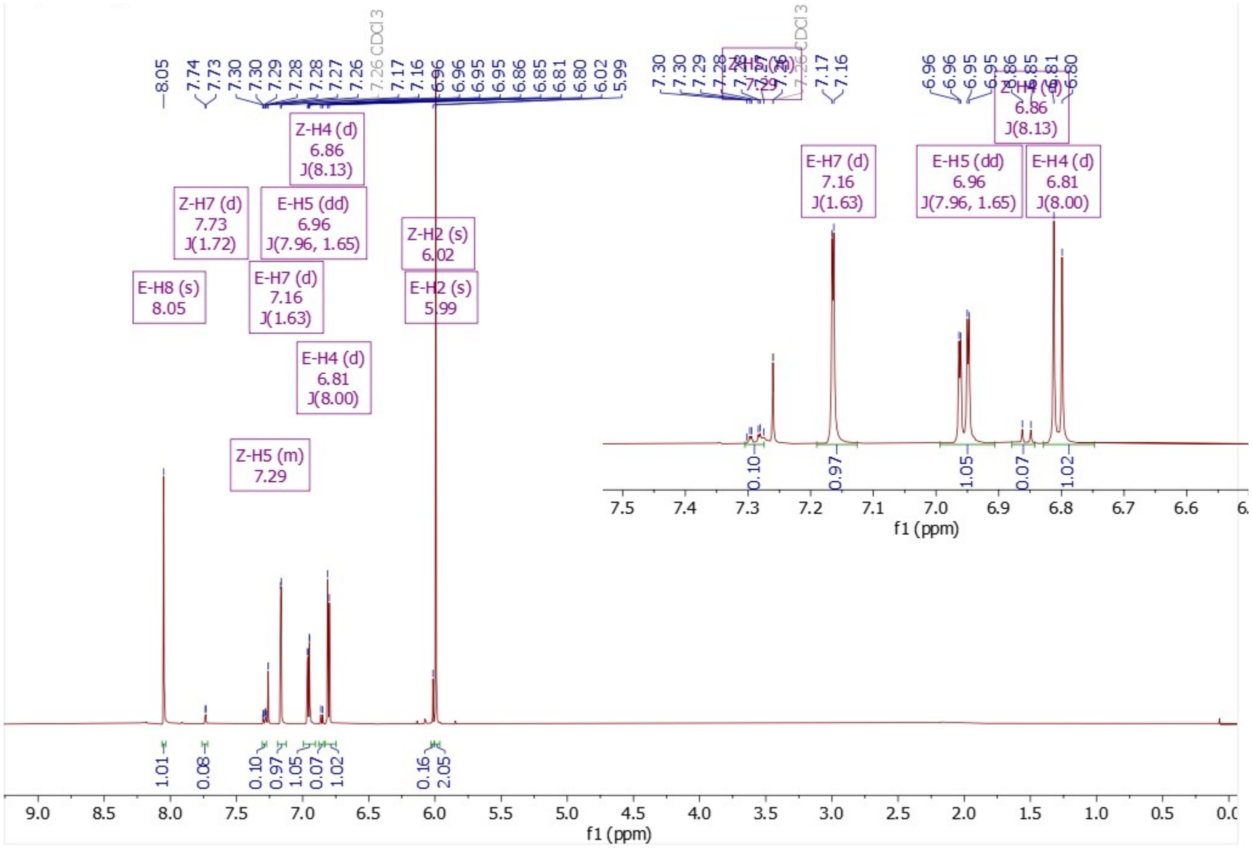

*2.13 Cinnamaldehyde oxime (**4c**) synthesis and purification*

Synthesis was performed according to Hansen *et al.*[6] Sodium hydroxide (0.647 g, 16.48 mmol, 1.05 equiv.) was dissolved in butanol (10 mL), deionized water (30 mL) and ethanol (20 mL). Subsequently, hydroxylamine hydrochloride (1.08 g, 15.41 mmol, 1.00 equiv.) was dissolved in the mixture. Cinnamaldehyde (9.02 g, 90.1 mmol, 1.00 equiv.) was slowly added. After 3.5 h, 250 mg of hydroxylamine were added and additional 200 mg of hydroxylamine after 4.5 h. The reaction was stirred for 19 h at rt. TLC analysis confirmed full conversion (CY:EA (3:1), UV detection). 50 mL of deionized water was added. The aqueous solution was extracted with EA (3 x 100 mL). The organic layer was dried over Na_2SO_4 and concentrated to give a yellow, solid residue. The crude product was purified by flash chromatography (CY:EA,5:1,) to yield 1.25 g product with an *E*/*Z*-ratio of 21/79.

*2.14 Hexanal oxime (**5c**) synthesis and purification*

Hexanal oxime **5c** was synthesized according to Hinzmann et al.[7] **5b** was distilled prior to use. A RBF (250 mL) was charged with sodium carbonate (7.21 g, 68.0 mmol, 0.75 equiv.) was dissolved in water (142.5 mL) and ethanol (7.5 mL). Subsequently, hydroxylamine hydrochloride (9.54 g, 137 mmol, 1.50 equiv.) and **5b** (9.02 g, 90.1 mmol, 1.00 equiv.) were added. The reaction was stirred for 19 h at rt under argon atmosphere during which an oily layer was formed at the surface. TLC analysis (LP, stained with $KMnO_4$) confirmed full conversion. The aqueous solution was extracted with diethyl ether (3 x 100 mL). The organic layer was washed with brine (100 mL) and then dried over Na_2SO_4 and concentrated to yield a clear, oily residue. The crude product was purified by flash chromatography (CH_2Cl_2 + 1 % MeOH) and yielded 7.67 g of **5c** (74 %) as colorless crystals with an *E*/*Z*-ratio of 55/45.

^{1}H NMR (400 MHz, Chloroform-*d*) δ 0.56 – 1.05 (m, 6H, H6), 1.18 – 1.42 (m, 8H, H4, H5), 1.48 (dtd, *J* = 8.5, 7.1, 3.4 Hz, 4H, H3), 2.19 (dtd, *J* = 519.6, 7.5, 6.2 Hz, 2H, *E*-H2), 2.37 (td, *J* = 7.6, 5.4 Hz, 2H, *Z*-H2), 6.71 (t, *J* = 5.5 Hz, 1H, *Z*-H1), 7.42 (t, *J* = 6.1 Hz, 1H, *E*-H1), 9.00 (s, 2H, OH).

^{13}C NMR (101 MHz, Chloroform-*d*) δ 14.0 (d, C6), 22.5 (C5), 25.1 (*Z*-C2), 25.8 (*Z*-C3), 26.3 (*E*-C3), 29.6 (*E*-C2), 31.3 (*E*-C4), 31.6 (*Z*-C4), 152.4 (*E*-Z1), 153.0 (*Z*-C1).

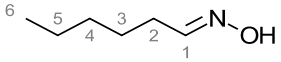

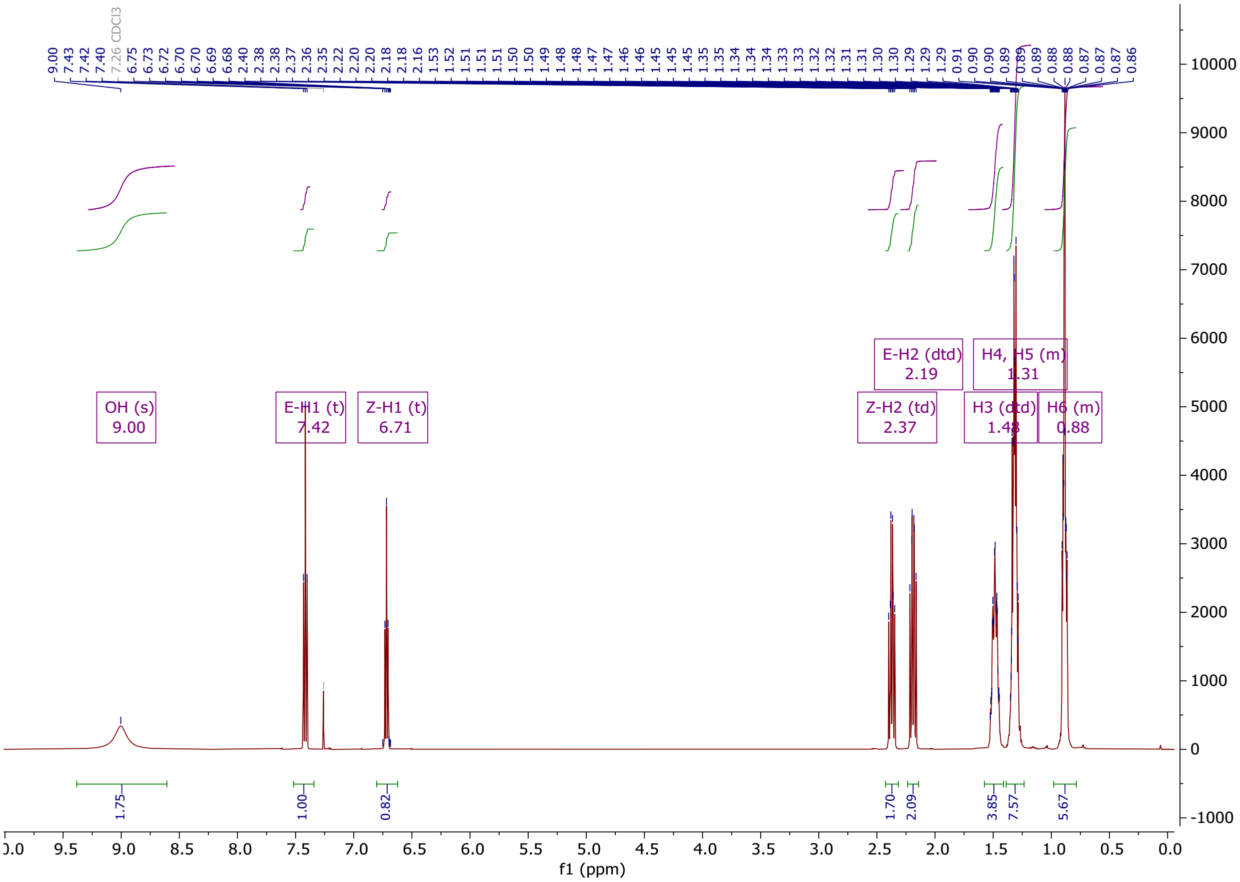

3. References

[1] R. Rädisch, M. Chmátal, L. Rucká, P. Novotný, L. Petrásková, P. Halada, M. Kotik, M. Pátek, L. Martínková, *Int. J. Biol. Macromol.* **2018**, *115*, 746–753.
[2] D. Schwendenwein, G. Fiume, H. Weber, F. Rudroff, M. Winkler, *Adv. Synth. Catal.* **2016**, *358*, 3414–3421.
[3] M. Horvat, S. Fritsche, R. Kourist, M. Winkler, *ChemCatChem* **2019**, *11*, 4171–4181.
[4] M. Horvat, T. S. Larch, F. Rudroff, M. Winkler, *Adv. Synth. Catal.* **2020**, *362*, 4673–4679.
[5] M. Horvat, G. Fiume, S. Fritsche, M. Winkler, *J. Biotechnol.* **2019**, *304*, 44–51.
[6] T. V. Hansen, P. Wu, V. V. Fokin, *J. Org. Chem.* **2005**, *70*, 7761–7764.
[7] A. Hinzmann, S. Glinski, M. Worm, H. Gröger, *J. Org. Chem.* **2019**, *84*, 4867–4872.
